# Structure and ligand binding of the ADP-binding domain of the NAD^+^ riboswitch

**DOI:** 10.1261/rna.074898.120

**Published:** 2020-07

**Authors:** Lin Huang, Jia Wang, David M.J. Lilley

**Affiliations:** Cancer Research UK Nucleic Acid Structure Research Group, MSI/WTB Complex, The University of Dundee, Dundee DD1 5EH, United Kingdom

**Keywords:** riboregulation, RNA structure, X-ray crystallography, metal ions, nucleotide binding

## Abstract

The *nad*A motif is the first known NAD^+^-dependent riboswitch, comprising two similar tandem bulged stem–loop structures. We have determined the structure of the 5′ domain 1 of the riboswitch. It has three coaxial helical segments, separated by an ACANCCCC bulge and by an internal loop, with a tertiary contact between them that includes two C:G base pairs. We have determined the structure with a number of ligands related to NADH, but in each case only the ADP moiety is observed. The adenosine adopts an *anti* conformation, forms multiple hydrogen bonds across the width of the sugar edge of the penultimate C:G base pair of the helix preceding the bulge, and the observed contacts have been confirmed by mutagenesis and calorimetry. Two divalent metal ions play a key structural role at the narrow neck of the bulge. One makes direct bonding contacts to the diphosphate moiety, locking it into position. Thus the nucleobase, ribose, and phosphate groups of the ADP moiety are all specifically recognized by the RNA. The NAD^+^ riboswitch is modular. Domain 1 is an ADP binding domain that may be ancient and could potentially be used in combination with other ligand binding motifs such as CoA.

## INTRODUCTION

Riboswitches are *cis*-acting elements that occur predominantly in the 5′-untranslated regions of bacterial mRNA to control gene expression (Roth and Breaker 2009; Serganov and Nudler 2013; Sherwood and Henkin 2016). The RNA folds to create a specific binding site for a small molecule such as a metabolite or ion so as to change the structure and thereby alter the rate of either transcription or translation of the downstream gene. In general, the gene product will be functionally related to the small molecule that binds the riboswitch, such as being an enzyme in its biosynthetic pathway.

**FIGURE 1.**
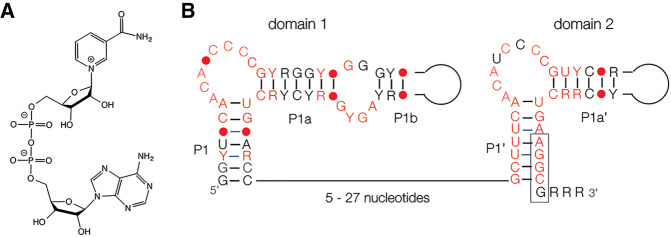
The structure of the coenzyme nicotinamide-adenine dinucleotide and the NAD^+^ riboswitch. (*A*) The chemical structure of NAD^+^, shown here in its oxidized form. (*B*) The sequence and secondary structure of the NAD^+^ riboswitch, adapted from Malkowski et al. (2019). Nucleotides conserved >97% are colored red, and the conserved nucleotide positions are shown by a red circle. The putative ribosome binding site is boxed.

Approximately 40 classes of riboswitches have been identified to date. While the biosynthesis of many coenzymes in bacteria is controlled by riboswitches, until recently none had been identified for the coenzyme nicotinamide-adenine dinucleotide (NAD). This is an important coenzyme that is involved in oxidation-reduction reactions in cellular metabolism (Fig. 1A), involved in transfer of electrons by switching between two oxidation states NAD^+^ and NADH. However, in the course of searching intergenic RNA sequences, Weinberg et al. (2017) identified a putative folded motif in the 5′-UTR of the *nad*A genes involved in NAD^+^ biosynthesis within the Acidobacteria phylum (Gazzaniga et al. 2009), that might act as a riboswitch. Very recently the Breaker laboratory have presented evidence that the *nad*A motif serves as an NAD^+^-dependent riboswitch to control expression of NAD^+^ biosynthetic genes (Malkowski et al. 2019). They identified over 100 unique sequences of this motif with an average length of 160 nt and deduced a consensus sequence and putative secondary structure.

Examination of the consensus sequence of the *nad*A motif reveals that it has two domains of similar sequence and secondary structure, each comprising a stem–loop with a conserved bulge of sequence ACANCCCC (Fig. 1B). Domain 1 consists of three helices P1, P1a, and P1b, where P1 and P1a are interrupted by the bulge, and P1a and P1b by a small conserved internal loop. Domain 2 is very similar, but lacks the internal loop, and has a putative ribosome binding site on the 3′ side of the P1′ helix. At first sight the tandem architecture is reminiscent of the glycine riboswitch that binds two molecules of glycine cooperatively to narrow the concentration range over which it switches (Mandal et al. 2004; Kwon and Strobel 2008; Butler et al. 2011; Baird and Ferre-D'Amare 2013). However, using in-line probing Malkowski et al. (2019) measured binding isotherms that provided no evidence for cooperative binding. Their probing experiments also indicated that only the ADP moiety of NADH (and ADP itself as well as a number of derivatives including AMP and ATP) bound to domain 1. In addition, they could find no evidence for binding of nicotinamide or other NAD^+^-related compounds to domain 2 despite its similarity in sequence to domain 1.

We therefore crystallized the NAD^+^ riboswitch domain I bound to NADH, ADP and other NAD^+^-related compounds, and solved their structures by X-ray crystallography. We find that helices P1, P1a, and P1b are coaxial, with a base paired interaction between the ACANCCCC bulge and the internal loop generating a pseudo-three-way junction that creates a binding site for ADP and related compounds. However, no interaction with the nicotinamide moiety of NAD^+^ or NADH can be observed in any complex. The adenine nucleoside of the ligand is hydrogen bonded to the riboswitch RNA, and the phosphate groups interact directly with one of two magnesium ions bound in the narrow neck of the ACANCCCC bulge.

## RESULTS

### Crystallization of the ADP-binding domain of the NAD^+^ riboswitch

A 52 nt RNA with the sequence of the *Candidatus koribacter versatilis* NAD^+^ riboswitch domain 1 was made by chemical synthesis and cocrystallized with a number of ligands related to NAD^+^ (Supplemental Table S1). 5-Bromocytosine was incorporated at position 3 or positions 42 and 45 in the P1 helix in order to provide phase information. These species crystallized in space group I222, with good resolution, and some of the structures were solved using SAD from the bromine atoms (Supplemental Table S2).

### The overall structure of the ADP-binding domain of the NAD^+^ riboswitch

**FIGURE 2.**
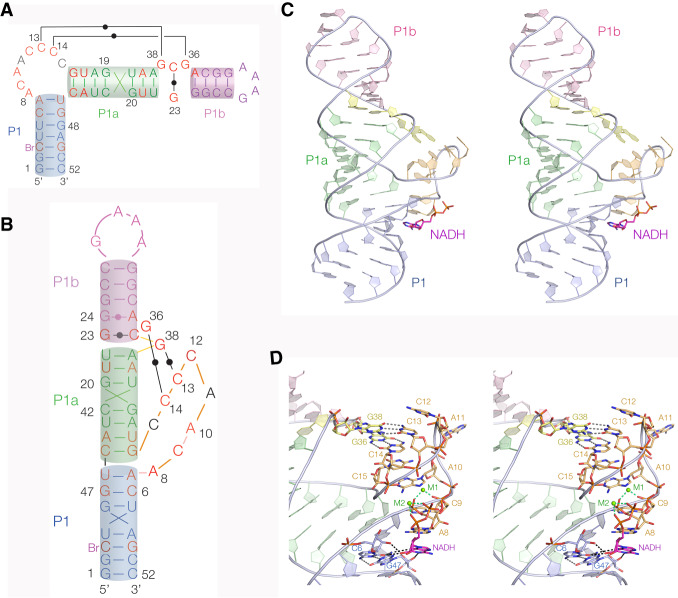
The overall structure of the NAD^+^ riboswitch domain 1 bound to NADH. (*A*) Secondary structure scheme drawn to correspond to that of Malkowski et al. (2019), using the color scheme followed in our images of the structure. Nucleotides conserved >97% are colored red. (*B*) Secondary structure scheme corresponding to that of our crystal structure. (*C*,*D*) Parallel-eye stereoscopic images of the crystal structure of domain 1. *C* shows an overall view of the structure with the RNA shown in cartoon form and the NADH ligand in stick form. *D* shows the bulge-loop interaction in detail.

The structure of domain 1 with bound NADH ligand is shown in Figure 2. Ligand binding is discussed in detail below, but we note that electron density is only visible for the ADP moiety of the NADH. The three helices P1, P1a, and P1b are coaxially stacked in the structure, effectively forming a single long helix. The conserved 8-nt ACANCCCC bulge (beginning at A8) at the internal end of P1 extends away from the helix. A8 and C9 are stacked on each other, A10 lies inside the bulge, while A11 is extended away from the body of the RNA. This is then followed by a sharp turn whereby the loop passes back toward the helix. The nucleobases of C12–C15 are stacked (the C_4_ stack), and C15 is also stacked on A10. Thereafter G16 is paired with C45 to form the first base pair of the P1a helix. Between P1a and P1b helices is a small internal loop containing a conserved GYG (beginning at G36) that lies on the opposite strand to the 8-nt bulge. C37 is base paired with G23, but G36 and G38 are base paired with C14 and C13, respectively, in the C_4_ stack of the 8-nt bulge. This explains why the length of the P1a helix is strongly conserved at 7 bp, as this is required to place the GYG and C_4_ sequences on the same side of the helix in order to form the two cross-strand base pairs. Furthermore, the great majority of the conserved nucleotides are contained within the bulged region and the interacting P1a–P1b loop (Supplemental Fig. S1).

**FIGURE 3.**
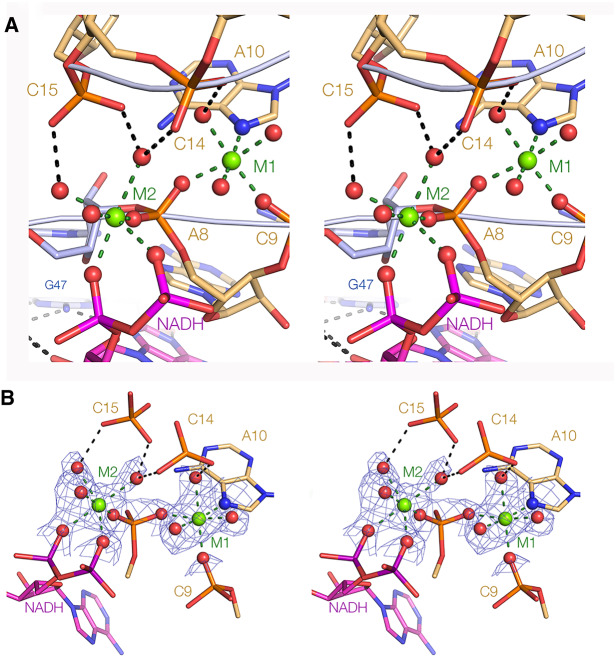
The narrow neck of the loop and the binding of divalent metal ions. (*A*) The close approximation of the two backbones and the binding of the two metal ions that span between the backbones, making both inner sphere and water-mediated contacts. Ion M1 is directly bound to phosphate nonbridging oxygen atoms at A8 and C9, and the N7 of A10. Ion M2 is directly bound to the other nonbridging oxygen of A8 and to those of the α and β phosphates of the NADH ligand. (*B*) The details of the coordination of the two metal ions with the experimental phasing map shown for the metal ions and directly bound ligands, contoured at 2σ.

The 8-nt bulge loop has an extremely narrow neck, with the phosphates at nucleotides 7, 8, and 9 approaching those at 14 and 15 with an average distance of 5 Å. The *pro*R nonbridging oxygen atoms of A8 and C15 are just 3.9 Å apart, and the *pro*S oxygen atoms of A7 and C15 are separated by 4.4 Å. However, electrostatic repulsion between the strands will be reduced by the binding of a number of metal ions to the RNA in this region. The backbones of the 5′ and 3′ ends of the loop are bridged by hydrated metal ions making both inner and outer sphere interactions with the RNA and the phosphate groups of the ligand (Fig. 3A). The experimental phasing map shows unambiguous electron density corresponding to the metal ions and inner sphere coordinated water molecules (Fig. 3B; Supplemental Fig. S2). This is supported by electron density calculated from anomalous scatter from Mn^2+^ ions diffused into the crystals that is observed in the same locations (Supplemental Fig. S3). Metal ion M1 bonds directly to the *pro*R oxygen atoms of A8 and C9 plus N7 of A10. The inner sphere water molecule apical with the C9 *pro*R oxygen is hydrogen bonded to C14 *pr*oR, so bridging the two strands in the neck of the loop. Metal ion M2 is bonded directly to the conserved A8 *pro*S, and to nonbridging oxygen atoms of each phosphate group of the ADP moiety of NAD^+^. Inner sphere water molecules apical with these phosphate groups are hydrogen bonded to nonbridging phosphate oxygen atoms C14 *pro*R and both nonbridging oxygen atoms of C15, again bridging the strands across the narrow the neck of the loop. Both metal ions have octahedral geometry with an average bond length of 2.07 and 2.12 Å for M1 and M2, respectively. The average bond length over the two ions is 2.09 ± 0.01 Å, consistent with bound magnesium ions. Additional hydrated metal ions, some making inner sphere interactions, are observed at other locations in the domain 1 structure, including one that is directly bonded to G47 O6.

### Binding of the ADP moiety for the NAD^+^ riboswitch domain 1

Although we first crystallized the NAD^+^ riboswitch domain 1 with NAD^+^, we could only observe electron density for the ADP moiety, and no density was visible in the maps corresponding to the nicotinamide mononucleoside (NMN) moiety. This is consistent with the in-line probing observations of Malkowski et al. (2019) who concluded that domain 1 of the riboswitch did not interact with NMN, but only ADP. A second crystal structure of the riboswitch bound to ADP alone showed that ligand to be bound in a near-identical manner to that of the ADP of NAD^+^ (discussed below and shown in Fig. 6; Supplemental Fig. S5), with an all atom RMSD = 0.29 Å.

**FIGURE 4.**
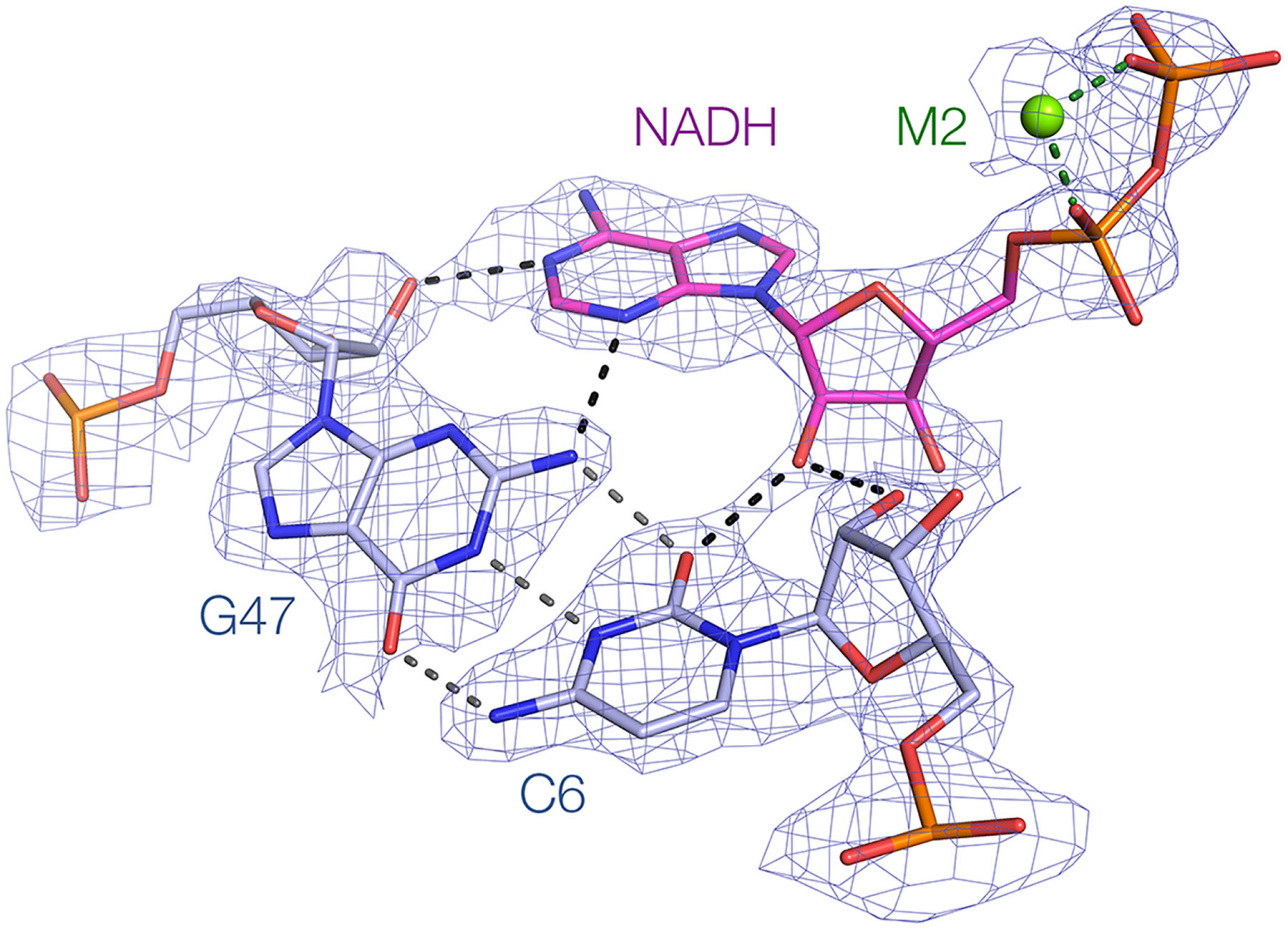
Hydrogen bonding of the ADP moiety of the NADH ligand to the minor groove side of the coplanar C6:G47 base pair. The experimental phasing map is shown contoured at 2σ. The *anti* conformation of the ADP moiety allows it to contact the entire width of the G:G base pair in the minor groove. The adenine forms a *trans* Watson–Crick sugar base pair with G47.

The ADP moiety adopts an *anti* conformation and is bound to the RNA in two distinct ways, via the adenosine section and via the phosphate groups. The 5′ end of the 8-nt bulge passing away from the P1 helix effectively extends the minor groove and forms a kind of pseudo-three-way junction that creates the binding site for the ligand. The sequence of this entire region is conserved (Supplemental Fig. S1). The adenine nucleobase of the ADP ligand is stacked with the nucleobase of A8, thereby forming a triple stack of ligand adenine-A8 and C9. The ligand nucleobase is coplanar with the conserved C6–G47 base pair of P1 (Fig. 4). The O2′ of the ligand is hydrogen-bonded to the O2′ (2.9 Å) and O2 (2.9 Å) of C6. The ligand adenine N3 accepts a hydrogen-bond from G47 exocyclic N2 (3.1 Å), and N1 accepts one from G47 O2′ (2.6 Å). This is essentially an A-minor interaction (Nissen et al. 2001). All the potential hydrogen bond donors and acceptors of the ligand adenosine are engaged except for N6 and N7 on the Hoogsteen edge that is directed away from the binding site, and the 3′-hydroxyl group. Malkowski et al. (2019) showed that *N*^6^-methyl-ATP and *N*^7^CH-ATP can both bind to the riboswitch, and we also demonstrate binding of the former by calorimetry and crystallography (see below); these observations are consistent with the Hoogsteen edge of the ligand adenine nucleobase making no direct interaction with the RNA.

The second part of the interaction of the ADP moiety with the domain 1 RNA involves the two phosphate groups. Oxygen atoms of the α (*pro*S atom) and β phosphates directly coordinate the M2 metal ion that is also directly bonded to A8 *pro*S oxygen, as well as by the hydrogen bonding of inner sphere water molecules to the C14 and 15 backbone of the strand on the other side of the neck (Fig. 3). These interactions will strongly locate the ADP phosphate groups in place, and corresponding interactions are found in all bound ADP derivatives (see below).

### Mutagenesis is fully consistent with the observed manner of ligand binding

**FIGURE 5.**
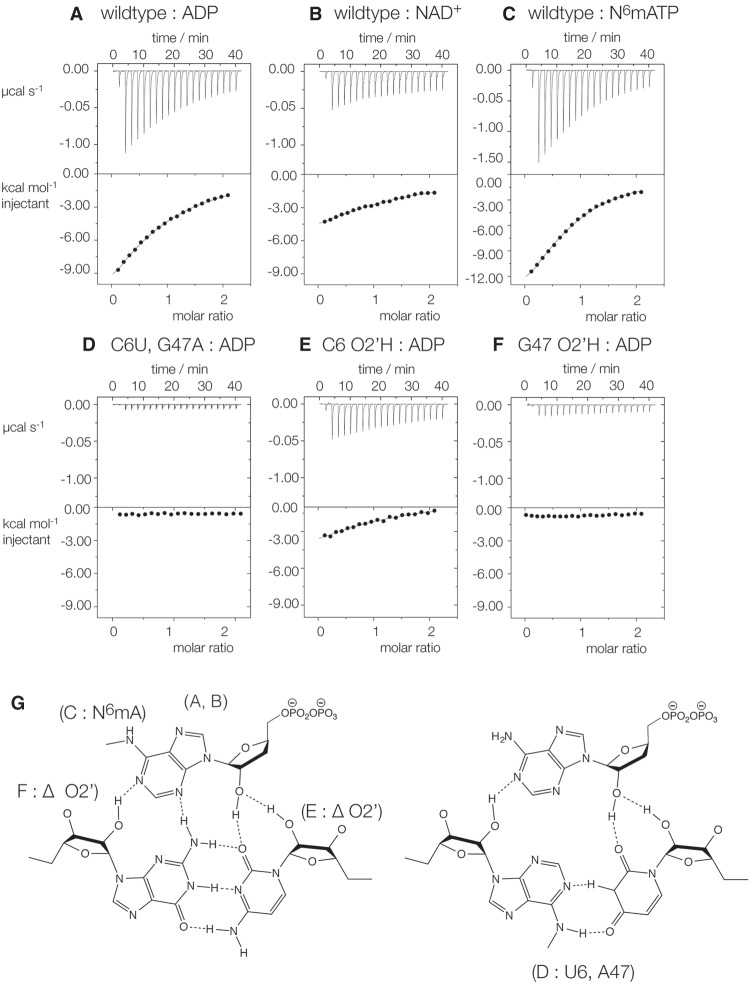
Ligand binding to the NAD^+^ riboswitch domain 1 analyzed by isothermal titration calorimetry and mutagenesis. A solution of NAD, ADP, or *N*^6^-methylATP was titrated into a wild-type or mutant NAD^+^ riboswitch domain 1 solution, and the heat evolved was measured as the power required to maintain zero temperature difference with a reference cell. Integration over time gives the heat required to maintain thermal equilibrium between cells. In each case, the *upper* panel shows the raw data for sequential injections of 2 µL volumes (following an initial injection of 0.4 µL) of a 1 mM solution of ligands into 200 µL of a 100 µM RNA solution in 40 mM HEPES (pH 7.2), 100 mM KCl, 10 mM MgCl_2_. This represents the differential of the total heat (i.e., enthalpy Δ*H*° under conditions of constant pressure) for each domain 1 concentration. Integrated heat data were analyzed using a one-set-of-sites model in MicroCal Origin following the manufacturer's instructions. The first data point was excluded in the analysis. All ITC experiments were repeated a total of three times. (*A*–*C*) Titration of ADP (*A*), NAD^+^ (*B*), and *N*^6^-methylATP (*C*) into unmodified domain 1 RNA. (*D*–*F*) Titration of ADP into modified domain 1 RNA; C6U:G47A mutant (*D*), and atomic mutants generated by removing the 2′-hydroxyl groups of C6 (*E*) and G47 (*F*). (*G*) Chemical structures of the interactions showing the mutations used in the ITC experiments.

We have tested the importance of the interactions between the ADP and the riboswitch domain 1 observed in the crystal by isothermal titration calorimetric (ITC) measurements of mutants of the RNA. Titration of ADP into the unmodified domain 1 RNA led to an exothermic binding reaction (Fig. 5A). Fitting the data gave a stoichiometry of 0.9 ± 2, and an affinity of *K*_d_ = 89 ± 15 µM (Supplemental Table S3). ITC experiments show slightly stronger binding of *N*^6^-methyl-ATP (*K*_d_ = 50 ± 19 µM) (Fig. 5C), and confirm the weaker binding of NAD^+^ (*K*_d_ > 150 µM) (Fig. 5A,B).

Removal of the O2′ of either C6 or G47 resulted in extremely impaired binding of ADP (Fig. 5E,F), consistent with the role of the hydrogen bonds to the O2′ and N1 of the ligand. We also investigated the role of the hydrogen bond between the ligand N3 and G47 N2 by making a double replacement of C6U, G47A, that is, replacing the C:G base pair by U:A. ITC revealed a total absence of binding of ADP to this mutant domain 1 (Fig. 5D), showing the importance of the ADP N3—G47 N2 hydrogen bond.

The ITC results are fully consistent with the mode of binding observed in the crystal.

### Binding of other ligands to the NAD^+^ riboswitch domain 1

**FIGURE 6.**
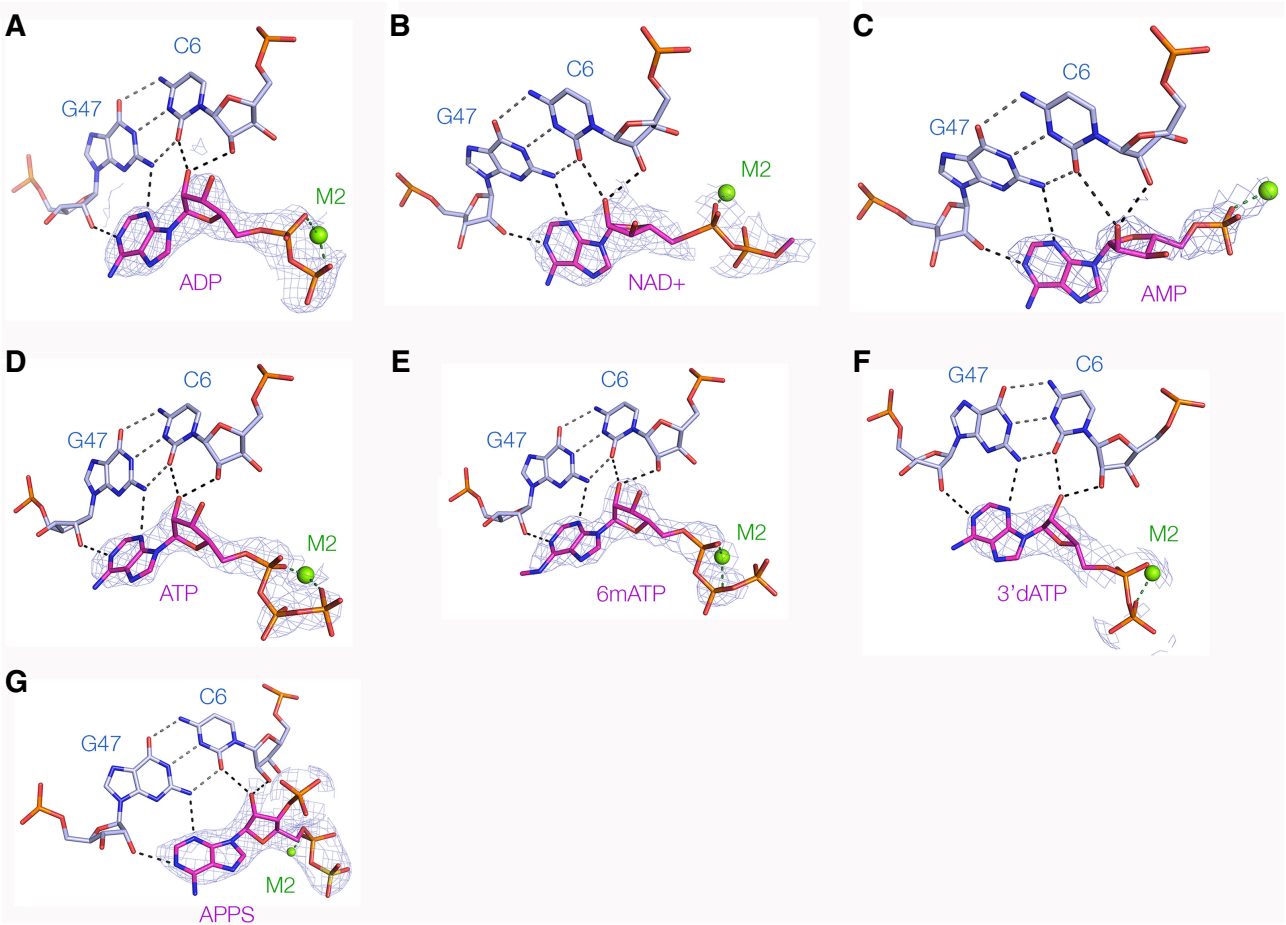
Binding of variant ligands to the NAD^+^ riboswitch domain 1. The RNA was cocrystallized with ligand variants and the structures solved either by SAD phasing or by molecular replacement using the structure bound to NADH (see Supplemental Table S1). For each structure is shown the C6:G47 base pair, metal M2, and the ligand with its electron density contoured at 2σ. The density maps are either omit maps (omit) or experimental phasing maps (exp). The ligands are: (*A*) ADP (omit); (*B*) NAD^+^ (omit), (*C*) AMP (exp); (*D*) ATP (exp); (*E*) *N*^6^-methylATP (exp); (*F*) 3′dATP (omit); (*G*) APPS (omit).

We have solved crystal structures of the NAD^+^ riboswitch domain 1 cocrystallized with a series of modified ligands related to ADP (Fig. 6; Supplemental Table S1). Malkowski et al. (2019) showed that a number of ADP derivatives bound to domain 1, and this provides the means to explore the generality of ligand binding by this RNA. For each ligand the structure of the RNA is essentially unaltered (Supplemental Fig. S4) with an average overall RMSD of 0.27 Å, and the manner of ligand binding is also closely similar (Supplemental Fig. S5). The binding of the adenosine moiety is essentially the same for each compound, with the adenine nucleobase stacked with A8 and its N1, N3, and O2′ hydrogen bonded to the RNA in an identical manner. Unsurprisingly the additional methyl group of *N*^6^-methylATP projects away from the RNA (Fig. 6E) and does not interfere with binding in agreement with our ITC measurements. The binding of APPS shows that a phosphate can be accommodated at the 3′ position (Fig. 6G). The phosphate groups all make very similar interactions with the M2 metal ion, in most cases nonbridging oxygen atoms of both α and β phosphates bonding directly to the metal atom. In the complex with ATP the γ phosphate also interacts with M2. Although electron density for only the ADP moiety is visible in the majority of the structures, in the NAD^+^ complex density is clearly present corresponding to the 5′ carbon atom of the nicotinamide moiety (Fig. 6B).

## DISCUSSION

It is probable that the *nad*A motif is an OFF riboswitch that regulates the biosynthesis of NAD^+^ in some bacteria (Malkowski et al. 2019). But the tandem character of the motif leaves a number of questions about the way the RNA might function as a riboswitch. The tandemly repeated glycine riboswitch binds two molecules of the amino acid cooperatively to give a sharper response to ligand binding (Mandal et al. 2004; Kwon and Strobel 2008; Butler et al. 2011; Baird and Ferre-D'Amare 2013). A tandemly repeated TPP riboswitch has been described that binds two molecules of thiamine pyrophosphate in a noncooperative manner (Welz and Breaker 2007). However, our ITC measurements clearly show that a single molecule of NADH binds to the isolated domain 1 of the NAD^+^ riboswitch, and our crystallographic studies show that this domain interacts with only half of NADH. Crystallographic analysis reveals that NAD^+^ and related compounds bind to the domain, but that electron density can only be observed for the adenine nucleotide moiety of the coenzyme, extending to the 5′ carbon atom of the nicotinamide moiety with NAD^+^ bound. But no density is visible for the nicotinamide moiety itself, in agreement with the conclusions from in-line probing experiments from Malkowski et al. (2019).

Determination of structures resulting from cocrystallization of domain 1 with eight different ligands clearly shows that this half of the riboswitch is an adenosine diphosphate binding domain, and the two phosphates that link the two 5′ carbon atoms of NADH are tightly held by their interactions with the metal ions in the neck of the bulge. It is therefore probable that domains 1 and 2 are in close proximity in the complete riboswitch, and that the nicotinamide moiety binds to domain 2. Domain 2 has a 7-nt bulge of closely similar sequence to that of domain 1, but lacks the internal loop corresponding to that between P1a and P1b in domain 1 so that the bulge in domain 2 is unlikely to make a similar tertiary base-pairing interaction. Yet the near-identity of the bulge sequence and its conservation suggests that it could generate a similar binding pocket to the adenosine diphosphate binding site of domain 1, and this would be the probable binding site for nicotinamide. In addition, Malkowski et al. (2019) have pointed out the 3′ end of the P1′ stem of domain 2 is a probable ribosome binding site, and thus this domain likely functions as the expression platform of the riboswitch.

Domain 1 is specialized for the binding of adenosine diphosphate, and it uses several strategies to achieve this such that the nucleobase, the ribose and the phosphate groups are all recognized. The ligand binding pocket is formed by the bulge, creating a pseudo-three-way junction. The adenine nucleobase is stacked against A8 at the base of the bulged loop (Fig. 2D), and coplanar with the C6:G47 base pair with the minor groove edges juxtaposed. The adenosine adopts an *anti* conformation and makes contacts along the width of the C6:G47 base pair in the minor groove. This allows extensive hydrogen bonding between the nucleobases and ribose 2′-OH of both the base pair and the ligand (Fig. 4), and the ITC measurements show that disruption of these interactions results in greatly weakened binding (Fig. 5).

Perhaps the most striking feature of the NAD^+^ riboswitch domain I is the key role played by divalent metal ions. These both stabilize the structure of the binding pocket, and specifically bind the phosphate groups of the ligand. Metal ions M1 and M2 stabilize the narrow neck of the bulge, with both ions making direct and water-mediated contacts to backbone nonbridging phosphate oxygen atoms. Indeed, both metal ions have exchanged three water molecules from their first spheres of hydration for RNA or adenine phosphate ligands. Unusually M2 is directly bonded to N7 of A10 within the bulge loop. Importantly, the α and β phosphates of ADP and other NAD^+^ derivatives are directly bonded to M2. Such inner-sphere interactions are relatively slow to exchange (Neely and Connick 1970), and should make a significant contribution to the binding, locking the charged phosphate groups into place. Moreover, since the two phosphate groups connect the ribose groups of adenine and nicotinamide in NAD^+^ and NADH, this interaction likely forms the interface between domains 1 and 2 in the complete riboswitch.

It is useful to compare the interaction of the ADP moiety in the NAD^+^ riboswitch domain I with the binding of adenosine derivatives in other riboswitches, including the SAM-I, SAM-III, SAM-V, cobalamine, cyclic di-AMP and cyclic-GAMP riboswitches (Supplemental Fig. S6). In all cases, the adenine nucleobase is stacked on RNA nucleobases on one or both faces, and forms a base pair involving at least two hydrogen bonds. However, the nature of these base pairs is different in every case. In SAM-1 (Montange and Batey 2006) and SAM-V (Huang and Lilley 2018) the Hoogsteen edge of the adenine forms a base pair with a U from the RNA, but these are *cis* and *trans* respectively. Moreover, while the adenosine adopts a *syn* conformation in SAM-1, it is *anti* in SAM-V. In SAM-III (Lu et al. 2008) the Watson–Crick edge of a *syn* adenine is hydrogen bonded to the sugar edge of a G in an *trans* base pair, while in the cobalamine riboswitch (Peselis and Serganov 2012) the Watson–Crick edge of an *anti* adenine is hydrogen bonded to the Hoogsteen edge of an A, also forming a *trans* base pair. The binding mode that comes closest to that of the NAD^+^ riboswitch is that observed in the cyclic di-AMP riboswitch (Gao and Serganov 2014; Jones and Ferre-D'Amare 2014; Ren and Patel 2014). This has a pseudo-symmetrical structure that binds two cyclic di-AMP molecules. Each of the four adenosine moieties is held in a related manner, that is in part closely similar to that seen in the NAD^+^ riboswitch, with essentially the same minor groove interaction between the sugar edge of the adenosine with the minor groove edge of a G:C base pair. But unlike the NAD^+^ riboswitch, the adenines in the cyclic di-AMP riboswitch are fully surrounded by RNA in a pocket so that in each case *N*^6^ and *N*^7^ of the adenine are hydrogen bonded to the backbone of the RNA. In marked contrast, in the cyclic GAMP sensing riboswitch (Ren et al. 2015) the adenine of the ligand forms a Hoogsteen:Watson–Crick base pair with an adenine nucleobase. These comparisons illustrate the plethora of strategies by which RNA can selectively bind adenine nucleotides. In the interaction of the ADP moiety of NADH and its derivatives with domain 1 of the NAD^+^ riboswitch both the nucleoside (Fig. 4) and the phosphates (Fig. 3) make important contributions to the binding. The only region of the ADP moiety that fails to interact with the RNA is the Hoogsteen edge of the nucleobase, explaining why *N*^6^-methylATP can bind normally.

From these and previous studies we conclude that the NAD^+^ riboswitch has a modular character, with domain 1 serving as a highly specific ADP-binding domain. At the present time we do not know where the nicotinamide moiety will bind, although Malkowski et al. (2019) plausibly suggested that domain 2 could serve as its binding site. This raises the question of whether it could be combined with variant domains 2 to bind different coenzymes such as CoA. Breaker and coworkers (Malkowski et al. 2019) have described a sequence variant of the *nad*A motif termed *ubi*B that also comprises two domains. The 5′ domain is very similar to the *nad*A domain I, with an 8-nt bulge separated from an internal loop by 7 bp, and a C:G base pair as the penultimate base pair of the bulge-proximal end of P1. In contrast, domain 2 is an uninterrupted 11 bp stem–loop structure, that includes a putative ribosome binding site at the 3′ end of P1′. It is conceivable that the two domains act together to bind another compound that includes an ADP moiety connected to a different functionality. Our structures indicate that the environment of the ADP O2′ is constrained, but that of the O3′ is relatively free. We have obtained a structure of domain 1 bound to APPS that has a 3′-phosphate (Fig. 6G), confirming that a phosphate group can be accommodated at the 3′ position. CoA has a 3′-phosphate group, so in principle domain 1 could serve as the ADP binding domain for this coenzyme. Thus the role of the *nad*A ADP-binding domain I could be more general, and not restricted to the NAD^+^ riboswitch. This suggests it may be an ancient nucleotide-binding domain in RNA.

## MATERIALS AND METHODS

### Riboswitch ligands

NADH (10107735001), NAD^+^ (N1636), ADP (A5285), ATP (A2383), AMP (A2252), 3′dATP and APPS (A1651) were all obtained from Sigma. *N*^6^-methylATP (NU-1101L) was obtained from Jena Bioscience.

### Sequence alignment and analysis

The NAD^+^ riboswitch sequences were taken from Rfam under accession RF03013. The sequences were visualized and analyzed by Jalview, and several RNA sequences were selected for crystallization trials based on length and conservation property. All sequence analysis was performed in Jalview, using the published alignment (Malkowski et al. 2019).

### RNA synthesis

The NAD^+^ riboswitch wild type has the sequence from *Candidatus koribacter versatilis* (all sequences written 5′ to 3′):

GGCUUCAACAACCCCGUAGGUUGGGCCGCAAGGCAGCGAAUCUACUGGAGCC

The RNA used for crystallization was (^Br^C = 5-bromocytidine):

unmodified: GGCUUCAACAACCCCGUAGGUUGGGCCGAAAGGCAGCGAAUCUACUGGAGCC
1 ^Br^C: GG(^Br^C)UUCAACAACCCCGUAGGUUGGGCCGAAAGGCAGCGAAUCUACUGGAGCC,
2 ^Br^C: GGCUUCAACAACCCCGUAGGUUGGGCCGAAAGGCAGCGAAU(^Br^C)(UA(^Br^C)(UGGAGCC

The RNA used for calorimetry had the wild-type sequence, and the C6U, G47A mutant and atomic mutants were derived from this sequence.

RNA oligonucleotides were synthesized using *t*-BDMS phosphoramidite chemistry (Beaucage and Caruthers 1981) as described in Wilson et al. (2001), implemented on an Applied Biosystems 394DNA/RNA synthesizer. RNA was synthesized using ribonucleotide phosphoramidites with 2′O-*tert*-butyldimethyl-silyl (*t*-BDMS) protection (Hakimelahi et al. 1981; Perreault et al. 1990) (Link Technologies). Oligonucleotides containing 5-bromocytidine (ChemGenes) were deprotected in a 25% ethanol/ammonia solution for 36 h at 20°C. All oligoribonucleotides were redissolved in 100 μL of anhydrous DMSO and 125 μL triethylamine trihydrofluoride (Sigma-Aldrich) to remove *t*-BDMS groups, and agitated at 65°C in the dark for 2.5 h. After cooling on ice for 10 min, the RNA was precipitated with 1 mL of butanol, washed once with 70% ethanol and suspended in double-distilled water.

RNA was further purified by gel electrophoresis in polyacrylamide under denaturing conditions in the presence of 7 M urea. The full-length RNA product was visualized by UV shadowing. The band was excised and electroeluted using an Elutrap Electroelution System (GE Healthcare) into 45 mM Tris-borate (pH 8.5), 5 mM EDTA buffer for 12 h at 150 V at 4°C. The RNA was precipitated with isopropanol, washed once with 70% ethanol, and suspended in water or ITC buffer (40 mM HEPES-K [pH 7.0], 100 mM KCl, 10 mM MgCl_2_).

### Crystallization, structure determination, and refinement

A solution of 0.5 mM NAD^+^ riboswitch RNA (52 nt) in 5 mM HEPES (pH 7.6), 100 mM KCl was heated to 95°C for 1 min. The solution was slowly cooled to 20°C and MgCl_2_ added to a final concentration of 5 mM. Ligands (Sigma-Aldrich) were added to a final concentration of 5 mM. Crystals were grown by hanging drop vapor diffusion at 7°C using drops prepared by mixing 1 μL of the RNA–ligand complex with 1 μL of a reservoir solution comprising 50 mM sodium cacodylate (pH 6.2-7.2) (precise solution conditions are given in Supplemental Table S1), 100 mM Mg acetate, 200 mM KCl and 10% (w/v) polyethylene glycol 3350. All structures were obtained by cocrystallization with the eight different ligands. Crystals appeared after 3 d. The crystals were transferred into mother liquid with 30% extra either polyethylene glycol 200 (v/v) or ethylene glycol (Supplemental Table S1), then flash frozen by mounting in nylon loops and plunging into liquid nitrogen. The NADH-domain 1 crystal was soaked with 100 mM MnCl_2_ for 30 min to obtain structure 6TFH.

Diffraction data were collected on beamline I24 or I03 of Diamond Light Source. Data were processed by XIA2 (Winter et al. 2018). The resolution cutoff for the data was determined by examining by CC1/2 and the density map (Karplus and Diederichs 2012). The structure was determined by Br-SAD by AutoSol in PHENIX suite, or molecular replacement using PHASER (McCoy et al. 2007) with the search model 6TF0. Crystals grew in space group I222_1_ with unit cell dimensions *a* = 57 Å, *b* = 59 Å, and *c* = 191 Å (Supplemental Table S2). Models were adjusted manually using Coot (Emsley et al. 2010) and subjected to several rounds of adjustment and optimization using Coot, phenix.refine, and PDB_REDO (Joosten et al. 2014). Ligand restraints for refinement of *N*^6^-methylATP coordinates were generated using PRODRG (Schuttelkopf and van Aalten 2004). Composite omit maps were calculated using PHENIX. Model geometry and the fit to electron-density maps were monitored with MOLPROBITY (Chen et al. 2010) and the validation tools in Coot. Atomic coordinates and structure factor amplitudes have been deposited with the PDB with accession code as listed in Supplemental Table S2. Crystallographic statistics are presented in Supplemental Table S2.

### Isothermal titration calorimetry

Titrations were performed at 298 K using an ITC-200 microcalorimeter (GE). RNA solutions at 100 μM were prepared by diluting concentrated stocks into binding buffer containing 40 mM HEPES-K (pH 7.0), 100 mM KCl, 10 mM MgCl_2_ (ITC buffer). Ligands were prepared in the same binding buffer at a concentration of 1 mM. The sample cell was filled with 200 µL of RNA. Ligands were injected in a volume of 0.4 µL for the first injection and 2 µL for the next 19 injections using a computer-controlled 40 µL microsyringe with an injection interval of 120 sec. Titration of ligands into the binding buffer or titration of the binding buffer into the RNA solution resulted in negligible evolution of heat. Integrated heat data were analyzed using a one-set-of-sites model in MicroCal Origin following the manufacturer's instructions. The first data point was excluded in the analysis. The binding parameters enthalpy Δ*H* (cal mol^−1^), association constant *K*_a_ (M^−1^) and *n* (bound ligands per RNA) were variables in the fit. The binding free energy Δ*G* and reaction entropy Δ*S* were calculated using the relationships Δ*G* = −RTlnK, where *R* = 1.987 cal mol^−1^ K^−1^, *T* = 298 K and Δ*G* = Δ*H* − TΔS. The dissociation constant *K*_d_ was calculated as 1/*K*_a_.

## DATA DEPOSITION

Atomic coordinates and structure factors for the reported crystal structures have been deposited with the Protein Data bank under accession numbers 6TF0, 6TB7, 6TF1, 6TF2, 6TF3, 6TFE, 6TFF, 6TFG, and 6TFH.

## SUPPLEMENTAL MATERIAL

Supplemental material is available for this article.

## Supplementary Material

Supplemental Material
