# Supplementary material for: Structure and ligand binding of the ADP-binding domain of the NAD+ riboswitch
Source: RNA. 2020 Jul;26(7):878–87. doi: 10.1261/rna.074898.120 (PMC7297122; doi:10.1261/rna.074898.120)
Supplement: Supplemental Material [file supp_26_7_878__index.html]

Structure and ligand binding of the ADP-binding domain of the NAD+ riboswitch — Supplemental Material 

# Structure and ligand binding of the ADP-binding domain of the NAD+ riboswitch

## Supplemental Material

- Supplemental\_Material.pdf
