# Supplementary material for: Structure and ligand binding of the ADP-binding domain of the NAD+ riboswitch
Source: RNA. 2020 Jul;26(7):878–87. doi: 10.1261/rna.074898.120 (PMC7297122; doi:10.1261/rna.074898.120)
Supplement: Supplemental Material [file supp_074898.120_Supplemental_Material.pdf]

Structure and ligand binding of the ADP-binding domain of the NAD^+ riboswitch

Lin Huang, Jia Wang and David M. J. Lilley

SUPPLEMENTARY INFORMATION

SUPPLEMENTARY FIGURES

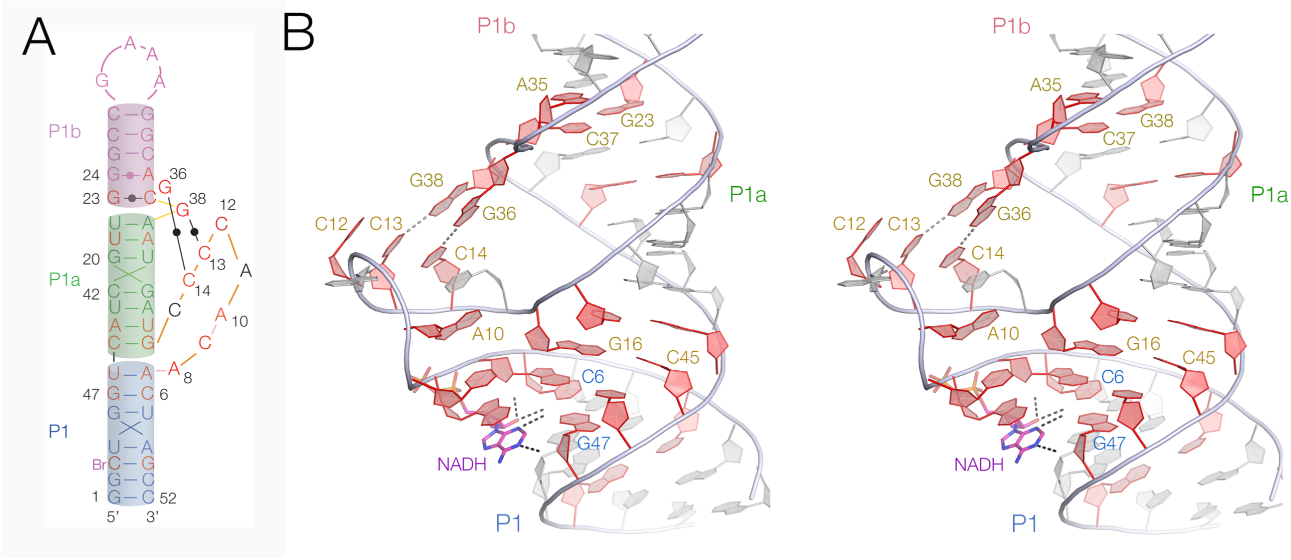

Figure S1. Positions of the conserved nucleotides shown in the structure of the NAD⁺ riboswitch domain 1. Nucleotides colored red at >97% conserved.

A. Scheme showing the structure of the RNA as found in the crystal.

B. Parallel-eye stereoscopic picture of the structure with the conserved nucleotides highlighted in red. Note that in this image the structure is viewed from the opposite site compared to that in Figure 1.

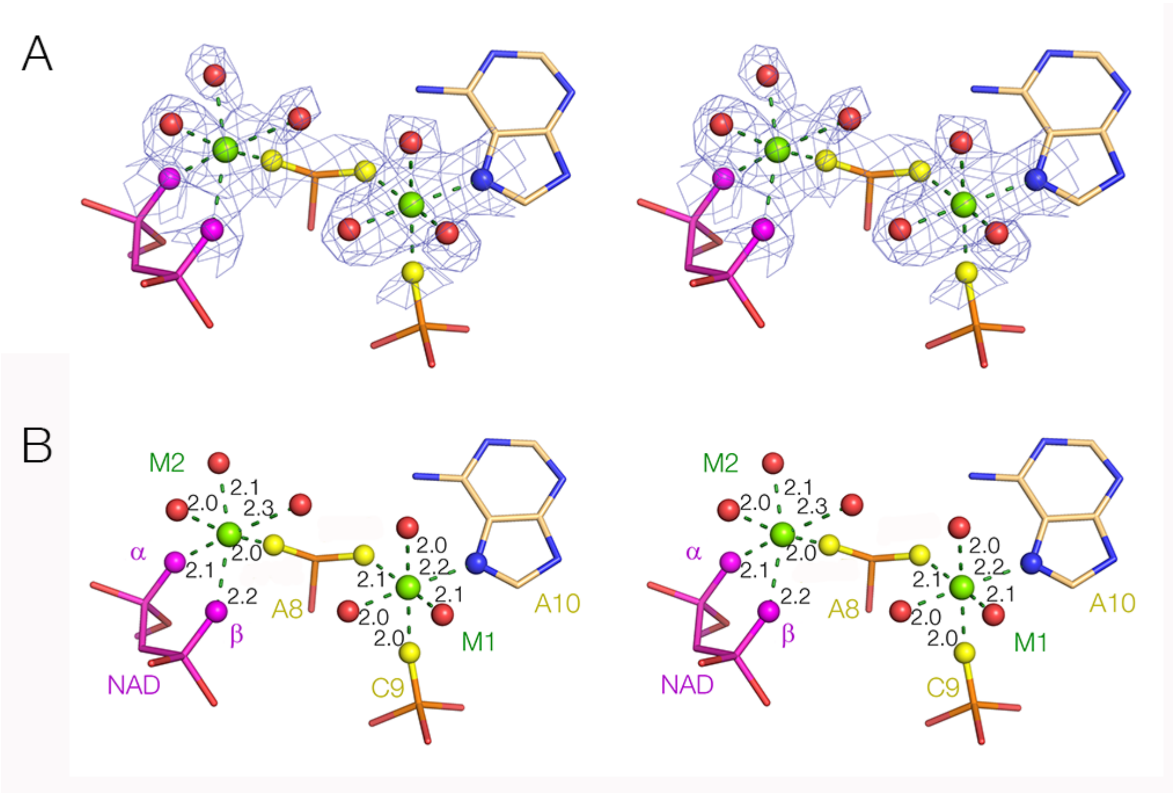

Figure S2. Coordination of metal ions M1 and M2 by the NAD$^+$ riboswitch domain 1 in the complex with NADH (PDB ID 6TF0). Parallel-eye stereoscopic views are shown.

A. The experimental phasing map for the two metal ions and their inner-sphere ligands contoured at 2 σ. Note that at this resolution (2.1 Å) the location of the ligands are unambiguous, and the octahedral coordination of the two metal ions is clear.

B. The same view with the ligands identified and the metal-ligand distances shown in Å. Ligand atoms are colored : blue = nitrogen; red = oxygen (water), yellow = oxygen (RNA phosphate), magenta = oxygen (NADH phosphate).

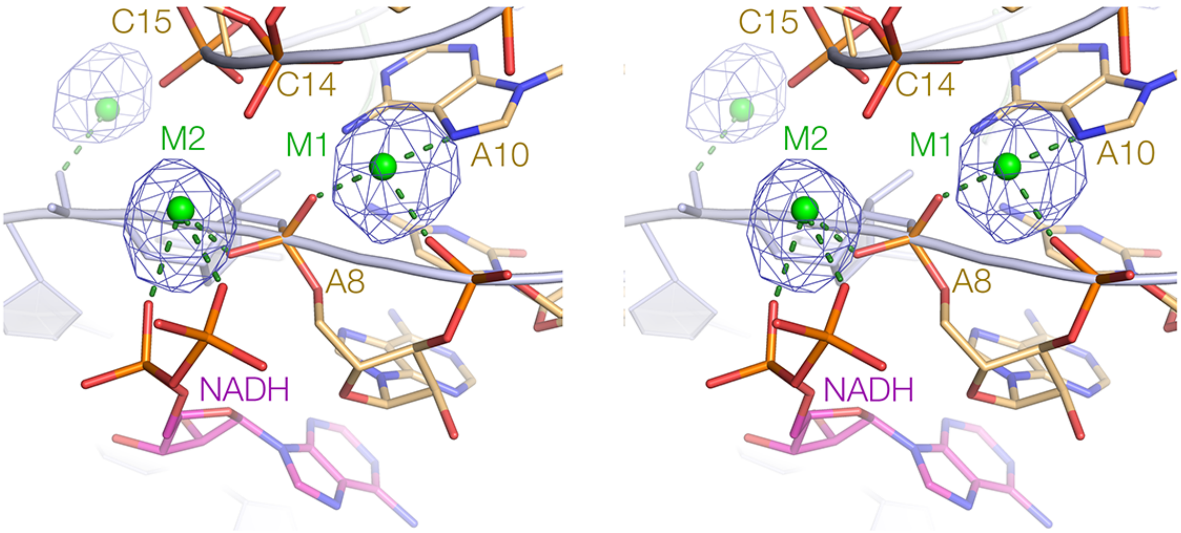

Figure S3. Manganese ions bound at the neck of the bulge of the NAD+ riboswitch domain 1. Parallel-eye stereoscopic image of the structure with the electron density from the anomalous scatter of Mn^{2+} ions shown contoured at 2 σ.

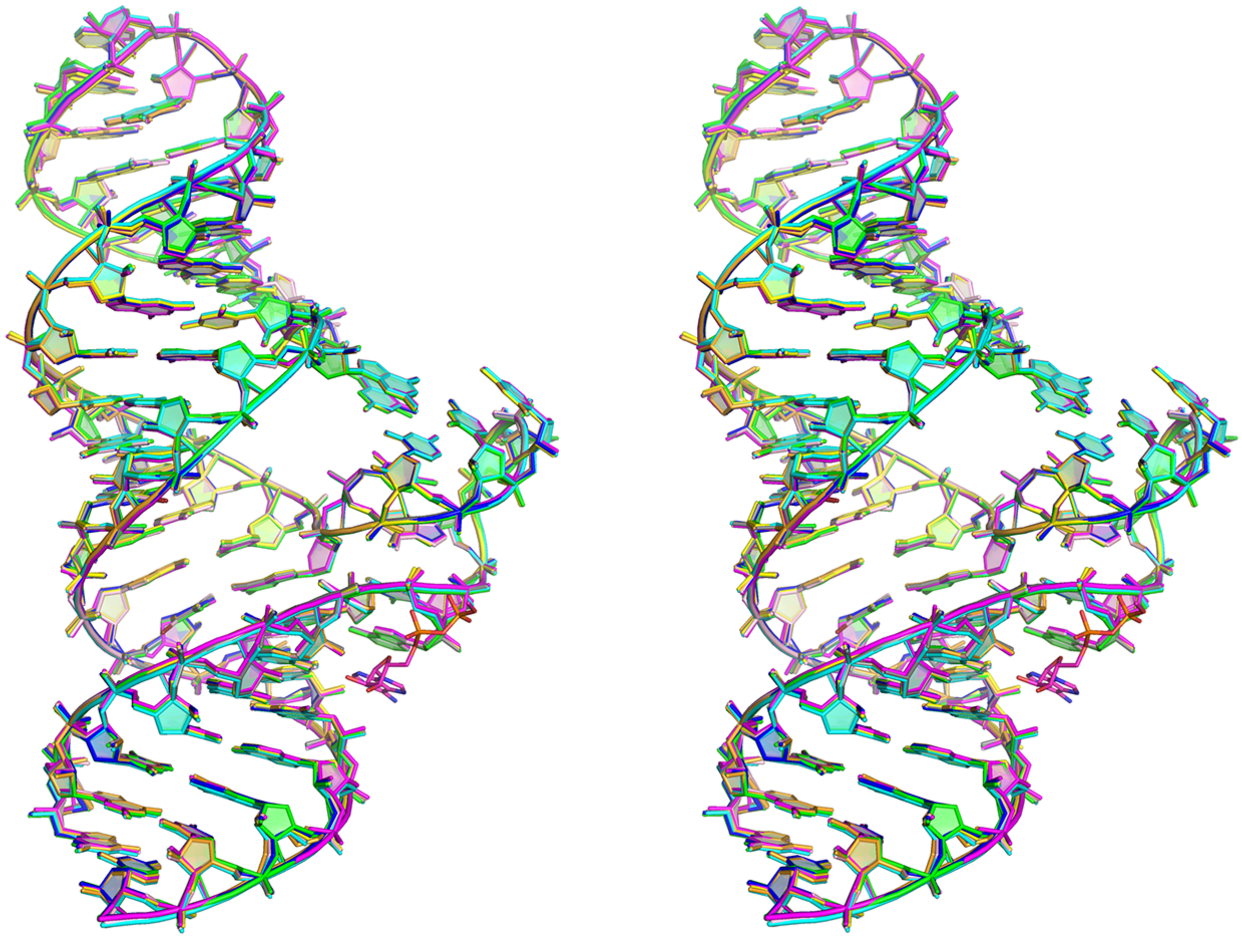

Figure S4. Parallel-eye stereoscopic image of a superposition of 8 structures of the NAD$^+$ riboswitch domain 1 with different bound ligands. NADH, green; NAD$^+$, cyan; AMP, blue; ADP, orange; ATP, pink; N^{6}mATP, yellow; APPS, grey; 3'dATP, magenta. Only the NADH ligand is shown in this image.

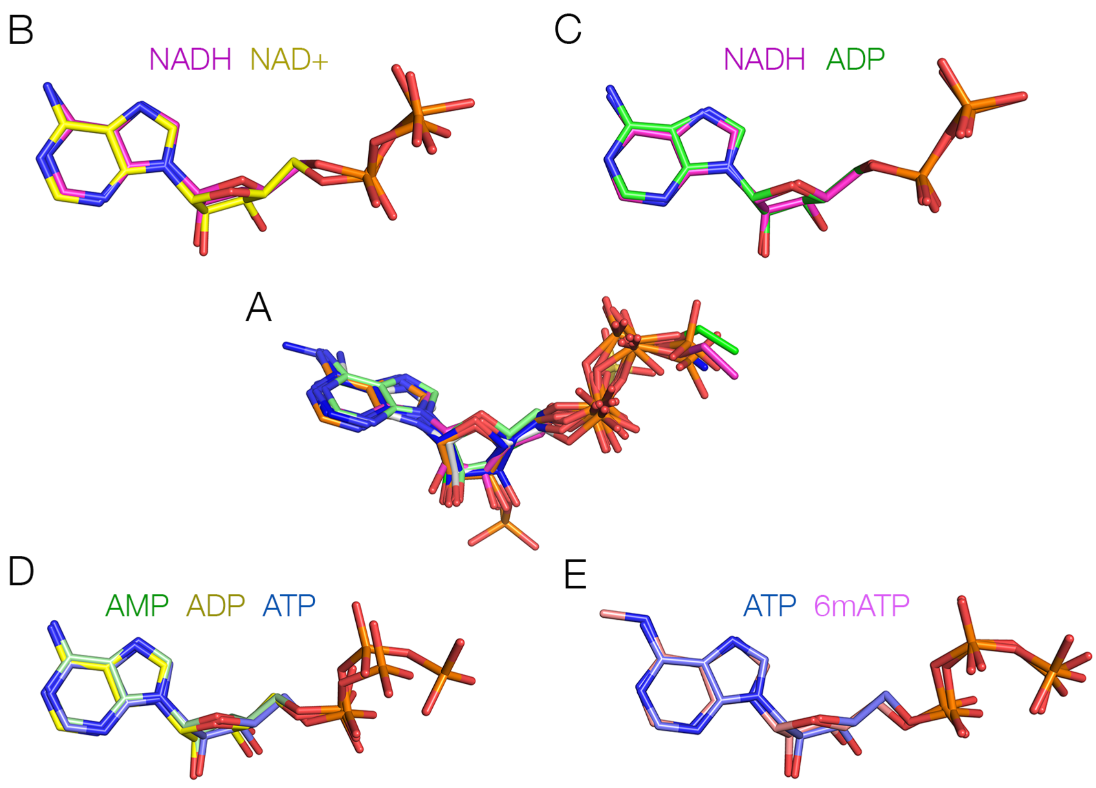

Figure S5. Superposition of ligand structures as observed bound to the NAD$^+$ riboswitch domain 1. These are taken from the overall superpositions shown in Figure S4.

A. Superposition of all eight ligands. NADH, NAD$^+$, AMP, ADP, ATP, N^{6}mATP, APPS and 3'dATP

B. Superposition of NADH (magenta) and NAD$^+$ (yellow).

C. Superposition of NADH (magenta) and ADP (green).

D. Superposition of AMP (green), ADP (yellow) and ATP (blue)

E. Superposition of ATP (blue) and N^{6}mATP (pink).

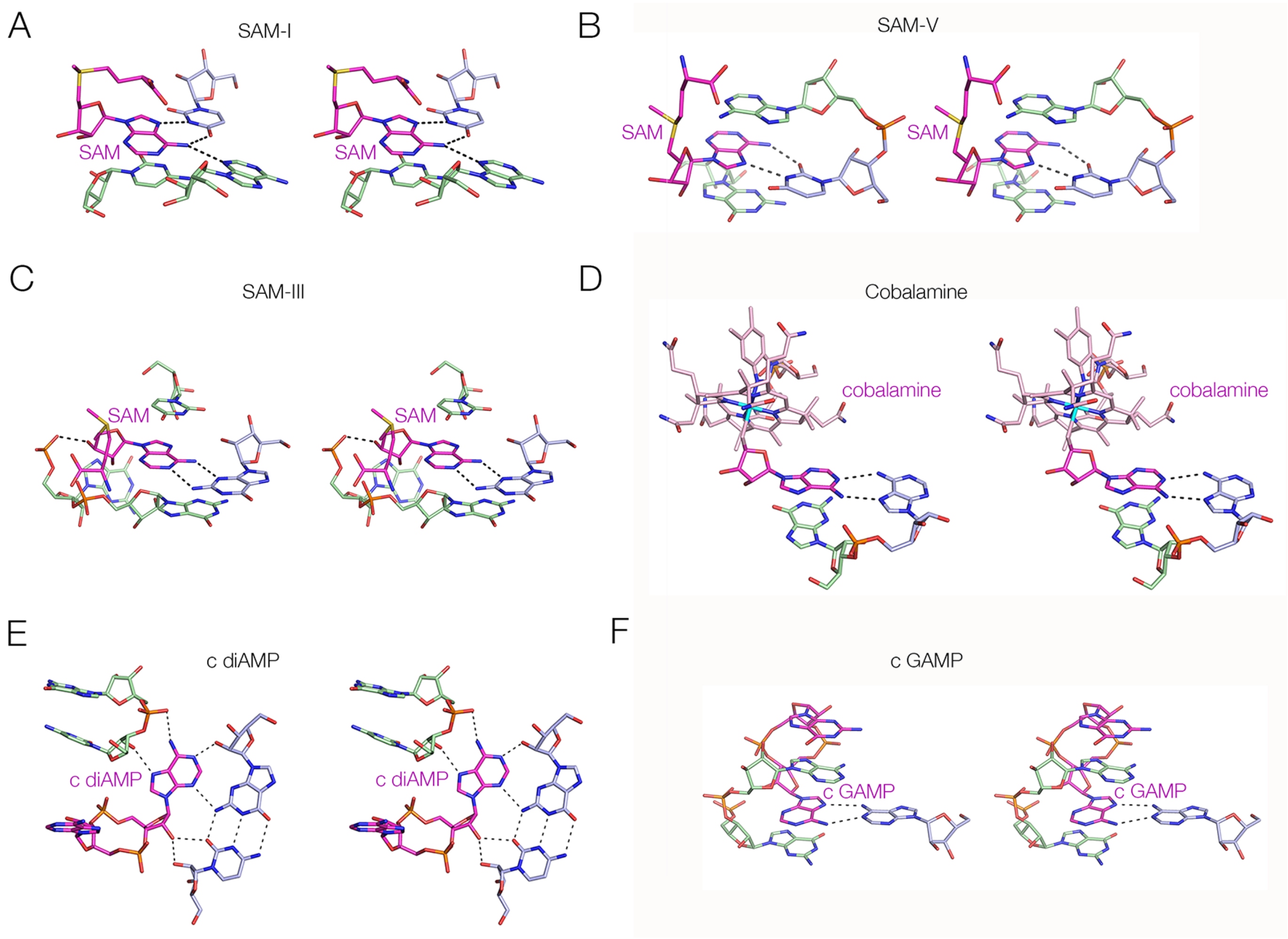

Figure S6. Comparison of the local interaction of adenosine with RNA in other riboswitches. The local environment of the adenosine moiety within SAM, cobalamine, cyclic di-AMP and cyclic GAMP bound to RNA, shown as parallel-eye stereoscopic images. The ligand is colored magenta, the RNA nucleotide to which the adenine is base paired is shown blue, and other nucleotides are colored green.

A. The SAM-I riboswitch (Montange and Batey 2006). The adenosine adopts a *syn* conformation, and forms a *cis* Hoogsteen-Watson Crick base pair with a uridine. PDB ID 3GX5.

B. The SAM-V riboswitch (Huang and Lilley 2018). The adenosine adopts an *anti* conformation, and forms a *trans* Hoogsteen-Watson Crick base pair with a uridine. PDB ID 6FZO.

C. The SAM-III riboswitch (Lu et al. 2008). The adenosine adopts a *syn* conformation, and forms a *trans* Watson Crick-sugar base pair with a guanine. PDB ID 3E5C.

D. The cobalamine riboswitch (Peselis and Serganov 2012). The adenosine adopts an *anti* conformation, and forms a *trans* Watson Crick- Hoogsteen base pair with a adenine. PDB ID 6FZO.

E. The cyclic di-AMP riboswitch (Gao and Serganov 2014; Jones and Ferre-D'Amare 2014; Ren and Patel 2014). The adenosine adopts an *anti* conformation, and forms a minor-groove triple interaction with a G:C base pair, while its Hoogsteen edge makes interaction with the backbone of another RNA section defining the binding pocket. PDB ID 4QK8.

F. The cyclic GAMP riboswitch (Ren et al. 2015). The adenosine adopts an *anti* conformation, and forms a *trans* Hoogsteen-Watson Crick base pair with an adenine. PDB ID 4YAZ.

SUPPLEMENTARY TABLES

PDB ID	ligand	mother liquor	cryo-protectant	temp / °C	resolution / Å	space group	beamline
6TF0	NADH	0.2 M KCl, 0.1 M Mg acetate<br>0.05 M Na cacodylate (pH 6.6)<br>10% w/v polyethylene glycol 3350	30% PEG200	7	2.10	I222	I03
6TB7	AMP	0.2 M KCl, 0.1 M Mg acetate<br>0.05 M Na cacodylate (pH 7.0)<br>10% w/v polyethylene glycol 3350	30% EG	7	2.53	I222	I24
6TF1	ADP	0.2 M KCl, 0.1 M Mg acetate<br>0.05 M Na cacodylate (pH 7.0)<br>10% w/v polyethylene glycol 3350	30% EG	7	2.4	I222	I24
6TF2	ATP	0.2 M KCl, 0.1 M Mg acetate<br>0.05 M Na cacodylate (pH 6.8)<br>10% w/v polyethylene glycol 3350	30% EG	7	2.55	I222	I24
6TF3	3'dATP	0.2 M KCl, 0.1 M Mg acetate<br>0.05 M Na cacodylate (pH 6.2)<br>10% w/v polyethylene glycol 3350	30% EG	7	2.66	I222	I24
6TFE	N^6mATP	0.2 M KCl, 0.1 M Mg acetate<br>0.05 M Na cacodylate (pH 7.2)<br>10% w/v polyethylene glycol 3350	30% EG	7	2.30	I222	I24
6TFF	NAD^+	0.2 M KCl, 0.1 M Mg acetate<br>0.05 M Na cacodylate (pH 6.6)<br>10% w/v polyethylene glycol 3350	30% EG	7	2.52	I222	I24
6TFG	APPS	0.2 M KCl, 0.1 M Mg acetate<br>0.05 M Na cacodylate (pH 6.8)<br>10% w/v polyethylene glycol 3350	30% EG	7	2.45	I222	I24
6TFH	NADH<br>100 mM<br>$MnCl_2$	0.2 M KCl, 0.1 M Mg acetate<br>0.05 M Na cacodylate (pH 6.8)<br>10% w/v polyethylene glycol 3350	30% PEG200	7	2.9	I222	I03

Table S1. Crystallization conditions, resolution, space group and data collection beamlines (all at Diamond light source).

ligands	NADH	AMP	ADP	ATP	3dATP	N6mATP	NAD^{+}	APPS	NADH Mn
PDB	6TF0	6TB7	6TF1	6TF2	6TF3	6TFE	6TFF	6TFG	6TFH
RNA(^{Br}C) number	1	1	0	2	0	2	2	0	1
**Data collection**									
Space group	I222	I222	I222	I222	I222	I222	I222	I222	I222
Cell dimensions									
a, b, c (Å)	57.3, 59.2, 191.1	57.4, 59.4, 190.4	57.6, 59.0, 190.6	57.4, 59.0, 191.0	57.5, 58.2, 191.1	57.4, 59.4, 190.1	57.9, 59.8, 192.0	57.5, 59.0, 191.0	56.9, 58.4, 194.0
α, β, γ (°)	90 90 90	90 90 90	90 90 90	90 90 90	90 90 90	90 90 90	90 90 90	90 90 90	90 90 90
	SAD-Br	SAD-Br	MR	SAD-Br	MR	SAD-Br	MR	MR	MR
	Br-Peak	*Br-Peak*		*Br-Peak*		*Br-Peak*			*Mn-Peak*
Wavelength	0.9195	0.9186	0.9186	0.9186	0.9186	0.9186	0.9186	0.9186	1.8923
Resolution (Å)	32.11 – 2.10 (2.12 – 2.10)	47.60 – 2.53 (2.57 – 2.53)	56.37 – 2.23 (2.27 – 2.23)	54.98 – 2.49 (2.53 – 2.49)	55.09 – 2.66 (2.71 – 2.66)	47.52 – 2.30 (2.34 – 2.30)	55.42 – 2.52 (2.56 – 2.52)	47.77 – 2.45 (2.49 – 2.45)	48.49 – 2.95 (3.00 – 2.95)
R_{merge}	0.038 (1.444)	0.053 (2.525)	0.087 (6.928)	0.054 (2.873)	0.071 (2.069)	0.051 (1.823)	0.078 (2.926)	0.038 (1.924)	0.122 (2.700)
I / σI	18.1 (1.2)	20.1 (0.8)	9.5 (0.2)	18.2 (0.7)	14.9 (1.0)	16.1 (1.0)	12.6 (0.6)	21.7 (1.0)	10.8 (0.5)
CC (1/2)	1.00 (0.79)	1.00 (0.48)	0.97 (0.78)	1.00 (0.35)	1.00 (0.49)	1.00 (0.59)	1.00 (0.41)	1.00 (0.53)	1.00 (0.48)
Completeness (%)	98.8 (98.2)	98.4 (97.7)	98.6 (96.4)	98.7 (99.1)	98.3 (100)	99.2 (99.9)	99.9 (100)	98.6 (99.8)	98.8 (98.2)
Redundancy	6.6 (6.9)	6.2 (6.4)	5.3 (4.8)	6.3 (6.5)	6.3 (6.6)	6.3 (6.7)	6.3 (6.5)	6.3 (6.5)	5.2 (5.2)
**Refinement**									
Resolution (Å)	27.43 – 2.10 (2.18 – 2.10)	42.58 – 2.53 (2.62 – 2.53)	55.12 – 2.40 (2.49 – 2.40)	54.98 – 2.55 (2.64 – 2.55)	55.09 – 2.66 (2.76 – 2.66)	47.47 – 2.30 (2.38 – 2.30)	55.42 – 2.52 (2.61 – 2.52)	47.76 – 2.45 (2.54 – 2.45)	42.73 – 2.95 (3.06 – 2.95)
No. reflections	19157 (1885)	11103 (1088)	12615 (1049)	10858 (1066)	9432 (940)	14745 (1431)	11558 (1090)	12211 (1205)	6785 (586)
R_{work} / R_{free}	0.217 / 0.262	0.207 / 0.247	0.215/ 0.245	0.211 / 0.238	0.212 / 0.241	0.258 / 0.272	0.213 / 0.225	0.193 / 0.225	0.219 / 0.252
No. atoms									
macromolecules	1111	1111	1111	1111	1111	1111	1111	1111	1111
ligands	37	31	35	39	34	48	36	42	40
solvent	25	17	18	4	2	25	9	18	
B-factors									
macromolecules	81.6	84.3	77.6	83.9	87.6	95.7	74.5	87.5	111.2
ligands	114.6	95.7	84.2	104.5	85.4	177.9	101.0	91.7	156.1
solvent	73.8	66.6	65.3	73.2	62.7	86.2	80.0	71.9	
R.m.s. deviations									
Bond lengths (Å)	0.009	0.007	0.008	0.007	0.006	0.006	0.004	0.004	0.012
Bond angles (°)	1.63	1.34	1.61	1.38	1.03	1.06	0.86	0.88	1.93

*Values in parentheses are for highest-resolution shell.

Table S2. Details of data collection and refinement statistics for the crystallographic data as deposited with the PDB.

ligand	*n*	ΔH kcal mol^{-1}	ΔS cal.K^{-1}mol^{-1}	ΔG kcal mol^{-1}	K_d µM	c*
ADP	0.9 ± 0.2	-17 ± 4	-39	-5.5	89 ± 15	1.1
N^{6}mATP	0.9 ± 0.1	-19 ± 3	-44	-5.8	50 ± 19	2.0

Table S3. Isothermal titration calorimetry of ADP and N^6-methylATP binding to the NAD$^+$ riboswitch domain 1 at 298 K.

References

Gao A, Serganov A. 2014. Structural insights into recognition of c-di-AMP by the ydaO riboswitch. *Nature Chem Biol* **10**: 787-792.

Huang L, Lilley DMJ. 2018. Structure and ligand binding of the SAM-V riboswitch. *Nucleic Acids Res* **46**: 6869-6879.

Jones CP, Ferre-D'Amare AR. 2014. Crystal structure of a c-di-AMP riboswitch reveals an internally pseudo-dimeric RNA. *EMBO J* **33**: 2692-2703.

Lu C, Smith AM, Fuchs RT, Ding F, Rajashankar K, Henkin TM, Ke A. 2008. Crystal structures of the SAM-III/S(MK) riboswitch reveal the SAM-dependent translation inhibition mechanism. *Nature Struct Mol Biol* **15**: 1076-1083.

Montange RK, Batey RT. 2006. Structure of the S-adenosylmethionine riboswitch regulatory mRNA element. *Nature* **441**: 1172-1175.

Peselis A, Serganov A. 2012. Structural insights into ligand binding and gene expression control by an adenosylcobalamin riboswitch. *Nature Struct Molec Biol* **19**: 1182-1184.

Ren AM, Patel DJ. 2014. c-di-AMP binds the ydaO riboswitch in two pseudo-symmetry-related pockets. *Nature Chem Biol* **10**: 780-786.

Ren AM, Wang XC, Kellenberger CA, Rajashankar KR, Jones RA, Hammond MC, Patel DJ. 2015. Structural basis for molecular discrimination by a 3 ',3 '-cGAMP sensing riboswitch. *Cell Rep* **11**: 671-671.
